# Region-specific inhibition of 14-3-3 proteins induces psychomotor behaviors in mice

**DOI:** 10.1038/s41537-018-0069-1

**Published:** 2019-01-14

**Authors:** Kourtney Graham, Jiajing Zhang, Haifa Qiao, Yuying Wu, Yi Zhou

**Affiliations:** 1 Department of Biomedical Sciences, Florida State University, College of Medicine, Tallahassee, FL 32306 USA; 2 Key Laboratory of Acupuncture and Herbs, Shaanxi University of Chinese Medicine, Century Ave, Xianyang Shi, Shaanxi Province, 712046 China

## Abstract

The 14-3-3 family of proteins is genetically linked to several psychiatric disorders, including schizophrenia. Our 14-3-3 functional knockout (FKO) mice, as well as other 14-3-3 knockout models, have been shown to exhibit behavioral endophenotypes related to schizophrenia. While specific forebrain regions, such as the prefrontal cortex (PFC) and hippocampus (HP), have been implicated in schizophrenic pathophysiology, the role of these brain regions in the top-down control of specific schizophrenia-associated behaviors has not been examined. Here, we used an adeno-associated virus (AAV) delivered shRNA to knock down the expression of the 14-3-3-inhibitor transgene, thus selectively restoring the function of 14-3-3 in the forebrain of the 14-3-3 FKO mice, we found that injection of the AAV-shRNA into both the PFC and the HP is necessary to attenuate psychomotor activity of the 14-3-3 FKO mice. Furthermore, we found that acute inhibition of 14-3-3, through the delivery of an AAV expressing the 14-3-3 inhibitor to both the PFC and HP, can trigger psychomotor agitation. Interestingly, when assessing the two brain regions separately, we determined that AAV-mediated expression of the 14-3-3 inhibitor specifically within the HP alone is sufficient to induce several behavioral deficits including hyperactivity, impaired associative learning and memory, and reduced sensorimotor gating. In addition, we show that post-synaptic NMDA receptor levels are regulated by acute 14-3-3 manipulations. Taken together, findings from this study directly link 14-3-3 inhibition in specific forebrain regions to certain schizophrenia-associated endophenotypes.

## Introduction

Schizophrenia is a debilitating psychiatric disorder characterized by a variety of symptoms encompassing multiple cognitive-behavioral domains, most of which can be attributed to deficits in higher-order brain functions. Consistently, postmortem analyses and MRI studies in schizophrenic patients have identified pathological changes in various forebrain regions, such as the prefrontal cortex (PFC) and hippocampus (HP).^1–4^ In addition, many functional MRI and PET studies of schizophrenic patients have revealed abnormal connectivity between the HP and PFC which are critical for the executive and cognitive functions.^4–9^ Thus, it is clear that functional alterations in these key forebrain regions are one of the core pathological features of schizophrenia. However, the role of specific forebrain regions in the circuit control of schizophrenic symptoms remains largely unknown.^10^

As human genetic studies identified a number of risk genes associated with schizophrenia, genetic animal models have become an increasingly valuable tool in addressing pathophysiological changes in schizophrenia at the molecular, synaptic, and circuitry levels.^11,12^ Previously, we and others have generated several animal models that target one of these candidate risk genes encoding 14-3-3, which refers to a family of ubiquitous proteins abundantly expressed in the brain.^13–15^ The 14-3-3 proteins are highly conserved from yeast to humans and consist of seven distinct isoforms (β,γ,ε,η,ζ,σ, and τ) in mammals.^16,17^ Single nucleotide polymorphisms (SNPs) of several 14-3-3 isoforms were revealed in various schizophrenic populations by linkage analyses.^18–21^ Ywhah, encoding the 14-3-3η isoform, is located within the established 22q12–13 candidate risk chromosomal region.^22^ Decreased expression of 14-3-3 at both the protein and mRNA levels was also detected in the brains of schizophrenic patients.^23–25^ More recently, Ywhag and Ywhaz, encoding 14-3-3γ and ζ isoforms, were identified as members of a group of glutamatergic postsynaptic proteins that are prone to de novo mutations in schizophrenic populations.^26–28^ Our previous 14-3-3 mouse model is considered to be a functional knockout (FKO) because it transgenically expresses a peptide inhibitor that antagonizes the binding of 14-3-3 proteins to their endogenous partners in an isoform-independent manner, thereby disrupting 14-3-3 functions in the brain.^29^ We found that inhibition of 14-3-3 in the brain results in a loss of dendritic spines and deficits in synaptic transmissions as well as behavioral abnormalities including cognitive deficits, locomotor hyperactivity, reduced sensorimotor gating, and social interaction deficiencies.^15,30^ These 14-3-3 FKO mice thus recapitulate schizophrenic-like endophenotypes, enabling us to further investigate the neurobiological basis of specific behavioral deficits.

In the 14-3-3 FKO mice, transgene expression is driven by the neuronal specific Thy-1 promoter that produces founder line-specific transgene expression patterns in the brain.^31^ Previous studies on different founder lines of 14-3-3 FKO mouse model suggest that the schizophrenia-associated behavioral abnormalities are correlated with expression of the 14-3-3 inhibitor transgene in the forebrain area.^15^ Here, we used an AAV-delivered shRNA that knocks-down the 14-3-3-inhibitor transgene expression to determine whether restoration of 14-3-3 functions in certain key brain regions attenuates the psychomotor activity of the 14-3-3 FKO mice. Furthermore, we injected an AAV expressed 14-3-3-inhibitor into these brain regions of the WT mice, and assessed resulting behavioral changes. Together, these approaches allow us to assess the link between 14-3-3 dysfunction in certain forebrain regions and schizophrenia-associated behaviors.

## Results

### Region-specific knockdown of the transgene expression in 14-3-3 FKO mice attenuates hyperlocomotor activity

**Fig. 1:**
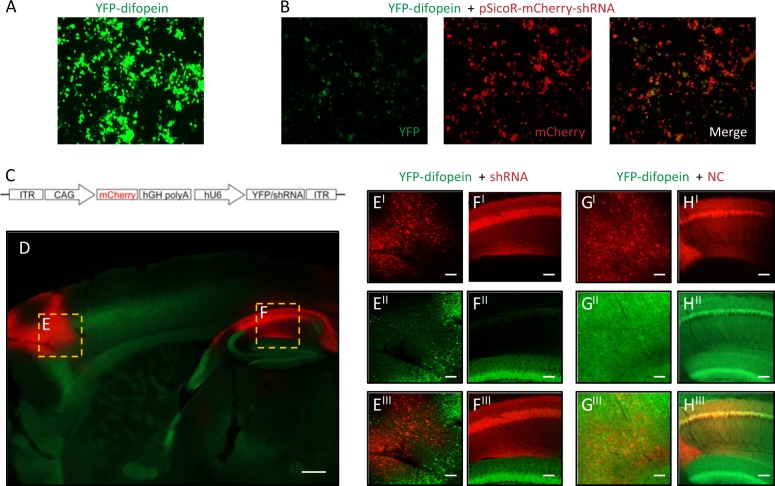
Region-specific knockdown of the transgene expression in 14-3-3 FKO mice. **a** Representative image of YFP (green) expression in tsA 201 cells transfected with YFP-difopein cDNA. **b** Representative images of reduced YFP expression in cells co-transfected with cDNAs of YFP-difopein and pSicoR-mCherry-shRNA. **c** Schematic map of recombinant adeno-associated viral (rAAV) vector used to co-express the shRNA against YFP (shRNA) and mCherry proteins. **d** Representative image of a sagittal section of a 14-3-3 FKO mouse brain injected with AAV-mCherry-shRNA (red) in both the prefrontal cortex (PFC) **e** and dorsal hippocampus (HP) **f**. Virus (red) and difopein transgene (green) expression were detected using fluorescent and confocal microscopy. There is minimal colocalization between red and green signals in the PFC (E^I−III^) or HP CA1 (F^I−III^), indicating a knockdown of difopein expression in AAV-infected cells. **g**, **h** Representative images of the PFC (G^I−III^) and the HP CA1 (H^I−III^) of the 14-3-3 FKO mice injected with the negative control (NC) AAV expressing a scrambled shRNA and mCherry. There is colocalization of red and green signals in both the PFC and HP regions. Scale bars represent 300 μm **d** and 100 μm **e**–**h**

Among all established 14-3-3 deficiency mouse models, the most consistent behavioral phenotype is novelty-induced hyperactivity, which is thought to correspond to positive or psychotic symptoms of schizophrenic patients.^32–34^ In order to understand the altered neural circuits underlying this abnormal behavior, we first asked whether selectively restoring the function of 14-3-3 proteins in certain brain regions could attenuate the hyperactive behaviors of the 14-3-3 FKO mice. Since the transgenically expressed 14-3-3 inhibitor difopein is fused with YFP, we reasoned that an shRNA against YFP would be effective in knocking down expression of the entire YFP-difopein fusion protein, thus restoring 14-3-3 functions in the 14-3-3 FKO mice. In vitro testing of multiple YFP-targeting shRNAs yielded one shRNA that was most effective in knocking down difopein expression (Fig. 1a, b). We then constructed an AAV with this shRNA sequence (AAV-mCherry-shRNA) and used this virus to restore 14-3-3 functions in specific brain regions of 14-3-3 FKO mice (Fig. 1c).

**Fig. 2:**
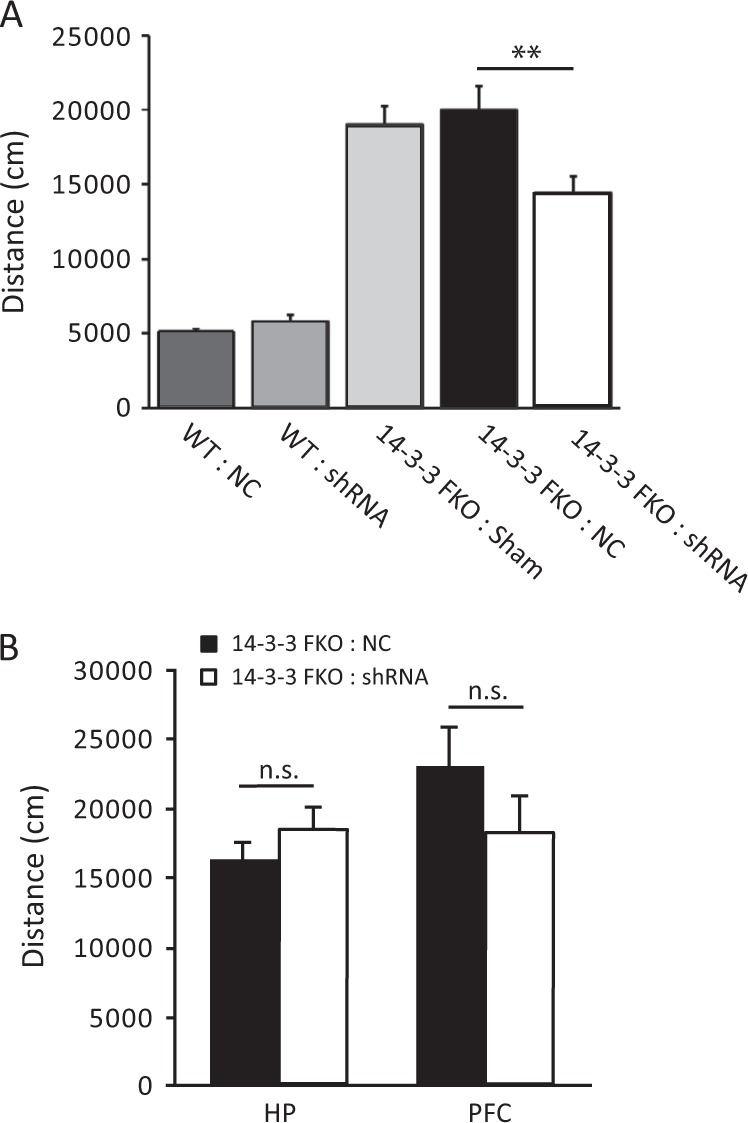
Attenuation of hyperlocomotive behavior in the 14-3-3 FKO mice. **a** Bilateral injection of the AVV-mCherry-YFP/shRNA to both the PFC and HP of 14-3-3 FKO mice (14-3-3 FKO: shRNA) (*n* = 12) significantly decreases distance traveled during open field testing, compared with 14-3-3 FKO mice injected with control virus injections in the same brain regions (14-3-3 FKO: NC) (*n* = 15; *F*(1, 25) = 12.57; *p* = 0.002). Distance traveled is similar in both WT mice with control virus injections (*n* = 6) and WT mice with shRNA injections (*n* = 10; *F*(1, 14) = 1.892; *p* = 0.191). **b** Bilateral injections of AAV-mCherry-YFP/shRNA to either the HP alone (*n* = 7; *F*(1, 10) = 0.836; *p* = 0.382) or PFC alone (*n* = 6; *F*(1, 10) = 1.516; *p* = 0.246) were not able to attenuate the locomotor activity in open field test when compared with 14-3-3 FKO mice injected with scrambled shRNA control (HP, *n* = 5; PFC, *n* = 6). Data are presented as mean ± S.E.M, with statistical significance denoted as: n.s., not significant; **p < 0.01; one-way ANOVA

In previous studies, we found that schizophrenia-associated behavioral deficits in 14-3-3 FKO mice correlate with high-level transgene expression in the forebrain region, particularly in the cortex and the HP.^15^ Thus, we bilaterally injected AAV-mCherry-shRNA into both the HP and PFC of the 14-3-3 FKO mice. As examined by fluorescent microscopy, the AAV-delivered shRNA effectively inhibited YFP-difopein expression in the HP and the PFC of the 14-3-3 FKO mice (Fig. 1d–h). Open field testing showed that their locomotor hyperactivity was significantly decreased following the injection of this shRNA virus (Fig. 2a). The attenuated hyperactivity of 14-3-3 FKO mice is likely mediated by shRNA knockdown of YFP-difopein, because bilateral injection of a scrambled shRNA (rAAV-mCherry-NC) into the two brain regions did not reduce the hyperactivity of 14-3-3 FKO mice, and neither AAV-mCherry- shRNA nor AAV-mCherry-NC had a significant effect on locomotor behavior of the WT mice with the same injections (Fig. 2a).

Furthermore, injection of AAV-mCherry-shRNA in only one of the brain regions, either the PFC or the HP, of the 14-3-3 FKO mice did not significantly decrease their hyperactivity in a 30-min open field test (Fig. 2b). Together, these data show that injection of shRNA to both the PFC and HP is necessary to attenuate hyperactivity in the 14-3-3 FKO mice, implicating the involvement of these forebrain regions in the top-down control of psychomotor behavior.

### Viral expression of the shRNA in specific brain regions increases the level of post-synaptic N-methyl-D-aspartate (NMDA) receptors

**Fig. 3:**
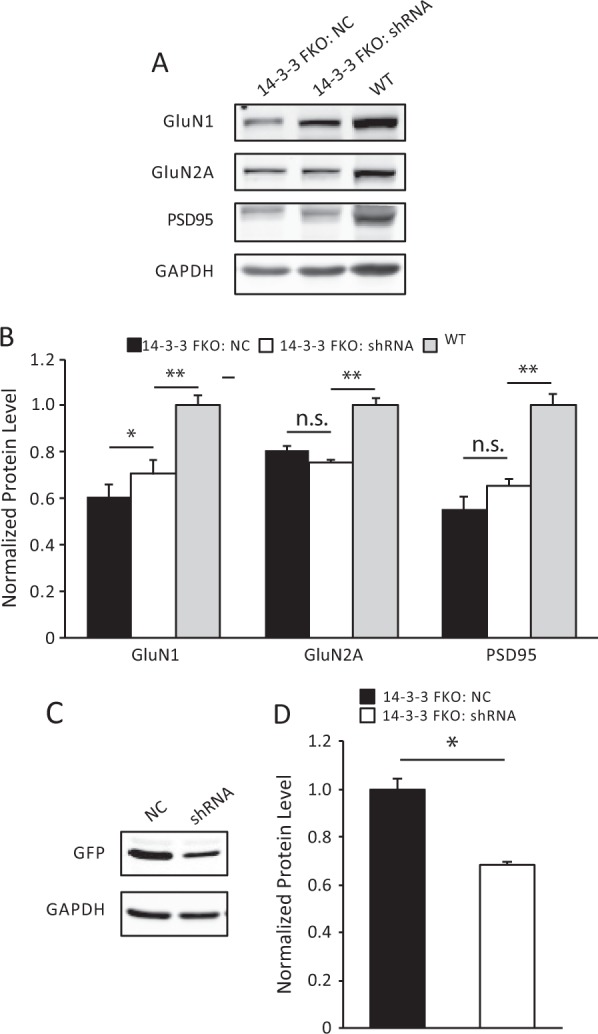
Elevated levels of post-synaptic density proteins induced by region-specific knockdown of the 14-3-3 inhibitor. **a**, **c** Representative images of western blots from dorsal hippocampal PSD fractions **a** and cell lysate **c** and after bilateral injections of AAV-mCherry-YFP/shRNA (shRNA) or the negative control AAV (NC) in the 14-3-3 FKO mice. **b** When compared with NC injections (*n* = 6), there is a significant increase in the GluN1 (*F*(1, 10) = 6.74, *p* = 0.048) subunit as well as an upward trend in PSD95 (*F*(1, 10) = 4.32, *p* = 0.09) in the PSD after bilateral injections of shRNA to the 14-3-3 FKO mice (*n* = 6). The levels of GluN1, GluN2A, and PSD95 in the shRNA group are lower than that in WT group (GluN1: *F*(1, 10) = 21.78, *p* = 0.006; GluN2A: *F*(1, 10) = 63.79, *p* < 0.001; PSD95: *F*(1, 10) = 29.87, *p* = 0.003). **d** AAV-mCherry-YFP/shRNA injections (*n* = 6) significantly reduced the levels of YFP-difopein in the dorsal hippocampus of 14-3-3 FKO mice, compared with controls (*n* = 6) (*F*(1, 10) = 14.54, *p* = 0.013). Blots were probed with GAPDH as a loading control. Protein levels were normalized to that of 14-3-3 FKO mice injected with control virus **b** or that of WT mice **d**. Data are presented as mean ± S.E.M., with statistical significance denoted as: n.s. = not significant, *p < 0.05, **p < 0.01; two-tailed *t*-test

We have shown previously that the levels of post-synaptic NMDA receptor subunits are significantly reduced in the forebrain of the 14-3-3 FKO mice.^30^ To test whether the reduced levels of post-synaptic NMDA receptor subunits could be rescued by knocking-down YFP-difopein expression in the forebrain, we measured the level of receptor subunits and synaptic proteins at the post-synaptic density (PSD) fraction (Fig. 3a, b) by western blotting. We found that the AAV-mCherry-shRNA injection to the HP of 14-3-3 FKO mice significantly increased the levels of GluN1 when compared with the 14-3-3 FKO mice that received negative control AAV injections. However, the shRNA virus did not completely restore the level of synaptic NMDA receptors to that of wild-type mice (Fig. 3a, b). Consistent with the partial rescue of NMDA receptors, the level of YFP-difopein expression in the HP was significantly reduced but not completely abolished with the shRNA virus injection when assessed by western blot analyses (Fig. 3c, d).

### Region-specific inhibition of 14-3-3 in WT mice results in hyperactive behavior and deficit in learning and memory

**Fig. 4:**
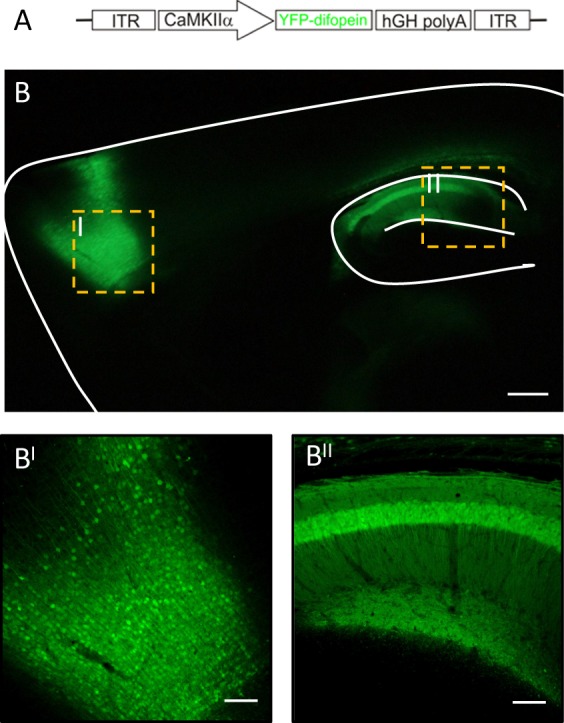
Region-specific expression of a 14-3-3 inhibitor in WT mice. **a** Schematic map of the recombinant adeno-associated viral (AAV) vector used to express the YFP-difopein under control of the CamKII promoter. **b** Representative images of a sagittal mouse brain section with YFP-difopein viral injections in both the PFC (B^I^) and dorsal HP (B^II^). YFP-difopein expression (green) was detected by fluorescent and confocal microscopy. Scale bars represent 300 and 100 μm respectively

Next, we sought to determine whether acute inhibition of 14-3-3 proteins in the HP and/or PFC is sufficient to induce schizophrenia-associated behavioral deficits in WT mice. As the transgene in 14-3-3 FKO mice is primarily expressed in excitatory neurons,^15,30^ we constructed another virus that uses the CamKII promoter to direct AAV-mediated expression of the 14-3-3 inhibitor, YFP-difopein (Fig. 4a, b).

**Fig. 5:**
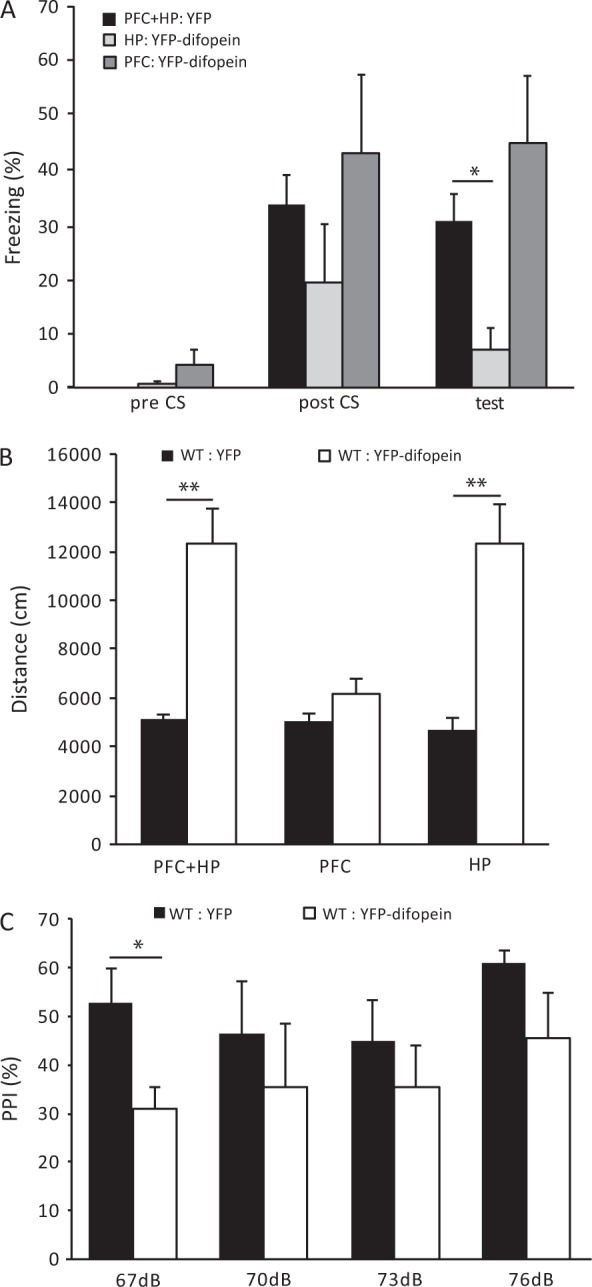
Behavioral deficits induced by YFP-difopein expression in WT mice. **a** Compared with WT mice injected with AAV-YFP in both the HP and PFC (*n* = 14), bilateral injections of AAV-YFP-difopein to the HP alone significantly reduce freezing behavior in contextual fear conditioning test 24 h after training (*n* = 6; *F*(1, 18) = 6.994; *p* = 0.016). However, injections of AAV-YFP-difopein to PFC alone does not affect freezing behavior when compared with WT controls (*n* = 6; *F*(1, 18) = 1.505; *p* = 0.236). **b** Bilateral injections of AAV-YFP-difopein into the HP, either alone (*n* = 8; *F*(1, 7.34) = 27.826; *p* = 0.001) or together with injection to the PFC (*n* = 9; *F*(1, 9.668) = 17.64; *p* = 0.002), significantly increase travel distances in open field test, compared with WT mice injected with AAV-YFP (HP: *n* = 9, HP + PFC: *n* = 6). However, injections of AAV-YFP-difopein to the PFC alone (*n* = 7; *F*(1, 10) = 1.953; *p* = 0.192) did not affect locomotive behavior of WT mice. **c** Bilateral injections of AAV-YFP-difopein to the HP of WT mice causes a decrease in pre-pulse inhibition (PPI) at all pre-pulse levels tested, while PPI is significantly lower at the pre-pulse level of 67 dB (*n* = 5; *F* (1, 8) = 6.746; *p* = 0.032), compared with WT mice with AAV-YFP injected to the HP of (*n* = 5). Data are presented as mean ± S.E.M. with statistical significance denoted as: *p < 0.05; **p < 0.01; one-way ANOVA

To evaluate the effectiveness of viral delivered 14-3-3 inhibitor expression, we first tested WT mice with AAV-YFP-difopein injection using contextual fear conditioning assay, which is known to be impaired by transgenic expression of the 14-3-3 inhibitor in the dorsal HP region.^30^ The freezing behavior was measured before training (Pre-CS), after training (Post-CS) and 24 h later (Test) (Fig. 5a). When compared with mice injected with the AAV-YFP control virus in both the HP and PFC, mice with bilateral injections of AAV-YFP-difopein into the HP had significant less freezing behavior during testing, suggesting a deficit in memory formation. In contrast, injections of YFP-difopein specifically to the PFC did not significantly decrease freezing behavior during testing (Fig. 5a). Thus, these results are consistent with our previous findings and validate the utility of region-specific 14-3-3 inhibition for assessing specific behavioral deficits.

We then utilized the AAV-mediated approach to express YFP-difopein in either the HP, PFC, or both regions in WT mice, and assessed their locomotor activity using an open field assay. Bilateral injection of AAV-YFP-difopein in both the PFC and dorsal HP of WT mice significantly increased locomotor activity during open field testing, when compared to that of WT mice with bilateral injection of a control virus (AAV-YFP) in both regions (Fig. 5b, left). Interestingly, we found that expression of YFP-difopein in the PFC alone had no effect on locomotor activity of the WT mice (Fig. 5b, middle). On the other hand, bilateral expression of YFP-difopein in only the dorsal HP of WT mice significantly increased distance traveled when compared with the control (Fig. 5b, right). Thus, it appears that inhibition of 14-3-3 in the dorsal HP has a more pronounced effect in inducing hyperactive behaviors, but both regions may play an important role in circuit control of this particular behavior.

In our previous study of the 14-3-3 FKO mice, we found that inhibition of 14-3-3 in the brain also results in reduced sensorimotor gating.^15^ Given that acute expression of the 14-3-3 inhibitor in the HP alone was sufficient to induce hyperlocomotive behavior and impair learning and memory, we further tested its effect on sensorimotor gating by measuring animals’ pre-pulse inhibition (PPI) to the acoustic startle response at four different pre-pulse levels (67, 70, 73, and 76 dB). Compared with WT mice with AAV-YFP injections, bilateral injection of AAV-YFP-difopein in the HP of WT mice resulted in a decrease in pre-pulse inhibition (Fig. 5c), suggesting a potential role of the HP in the regulation of sensorimotor gating.

### Viral expression of the 14-3-3 inhibitor reduces the level of post-synaptic NMDA receptors

**Fig. 6:**
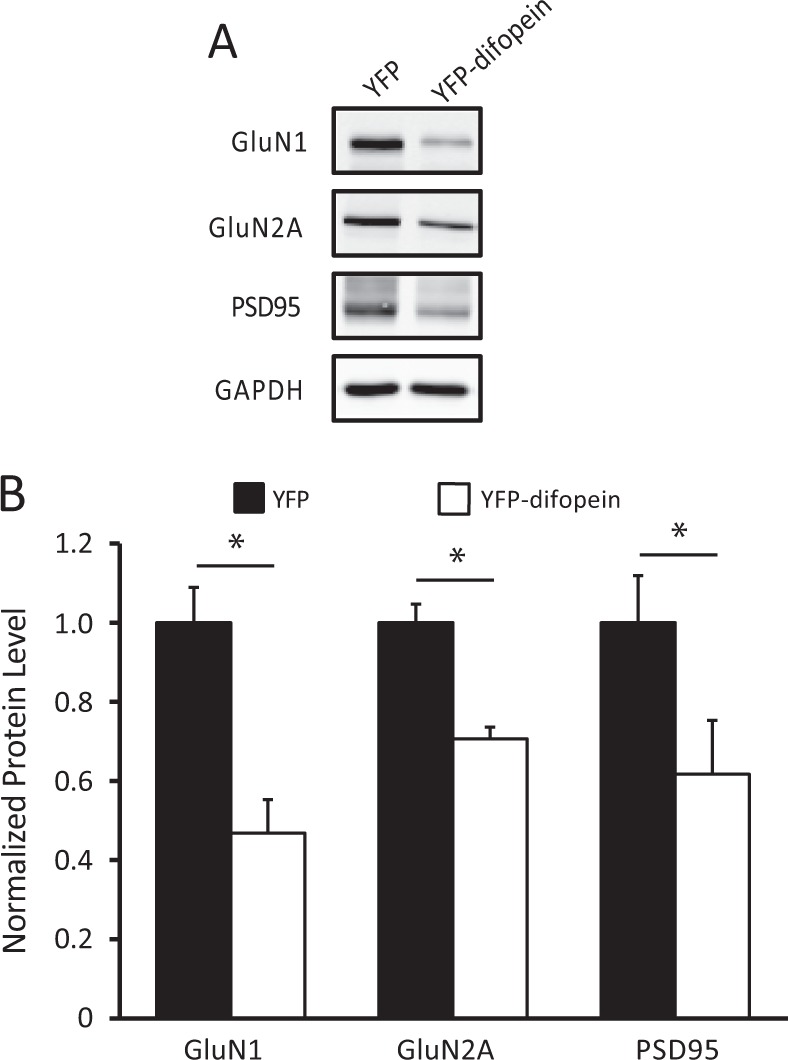
Reduction of NMDA receptor subunits in the post-synaptic density induced by YFP-difopein expression. **a** Representative images of western blots from dorsal hippocampal PSD fractions of WT mice injected with either control virus (AAV-YFP) or AAV-YFP-difopein virus. **b** Compared with that of controls (*n* = 6 for all groups), there is a significant decrease in the levels of GluN1 (*n* = 6, *F* (1, 10) = 14.60, *p* = 0.012), GluN2A (*n* = 6, *F*(1, 10) = 12.50, *p* = 0.017) subunits and the levels of PSD95 (*n* = 6, *F*(1, 10) = 11.73, *p* = 0.019) in YFP-difopein injected mice. Blots were probed with GAPDH as a loading control and protein levels were normalized to that of WT control. Data are presented as mean ± S.E.M., with statistical significance *p < 0.05, two-tailed *t*-test

Perinatal expression of the 14-3-3 inhibitor selectively reduces the NMDA currents in the Schaffer collateral to CA1 HP synapses, which correlates with decreased levels of NMDA receptors at the PSD.^30^ To determine if acute inhibition of 14-3-3 via AAV-YFP-difopein expression in adult WT mice is sufficient to induce similar changes, we measured the levels of NMDA receptor subunits in the PSD from the dorsal HP of the injected mice using western blot. In the WT mice injected with AAV-YFP-difopein, there was a significant reduction in levels of GluN1, GluN2A subunits and PSD 95, compared with WT mice injected with the AAV-YFP control virus (Fig. 6a, b). These results are consistent with the changes we have seen previously in the transgenic 14-3-3 FKO mice, suggesting that NMDA receptor regulation at the PSD is a direct effect of 14-3-3 inhibition.

## Discussion

This study follows our initial characterization of the 14-3-3 FKO mice, in which we identified mouse behavioral phenotypes that correspond to the cognitive, negative, and positive symptoms of schizophrenia.^15,30^ Clinically, a positive symptom of schizophrenia is defined as disorganized behavior that is usually modeled in rodents by novelty-induced hyperactivity. This endophenotype is thought to represent the underlying neural circuit abnormalities of positive symptoms, as hyperactivity in rodents can be induced by drugs that cause psychosis in humans and can also be ameliorated by antipsychotics.^32^ Based on our previous analyses of the 14-3-3 FKO mice, the psychomotor disturbance is likely due to disruption of 14-3-3 functions in the forebrain region.^15^ In the present study, we used a viral-mediated approach to define the key forebrain regions that are involved in 14-3-3 inhibition induced psychomotor behavior. By using AAV-mediated gene suppression or expression of the 14-3-3 inhibitor in 14-3-3FKO or WT mice respectively, we further examined whether 14-3-3 inhibition is necessary and/or sufficient to influence the psychomotor behavioral phenotype.

Positive symptoms of schizophrenia are caused by hyperactive dopaminergic signaling in the brain.^35,36^ As there is no apparent pathological alterations within the mesolimbic dopamine system of schizophrenic patients, the aberrant dopamine signaling and associated symptoms may be secondary to the dysfunction of cortical and hippocampal regions that regulate dopaminergic transmissions.^37,38^ Previously, we found a significant increase in the striatal DA content of the 14-3-3 FKO mice. Moreover, the heightened locomotor activity of these mice can be attenuated by antipsychotic drugs that mainly antagonize DA receptors.^15^ Similar findings were also reported for the 14-3-3ζ knockout mice.^34^ In this study, we found that inhibition of 14-3-3 proteins in the dorsal HP alone significantly increases locomotor activity in open field testing. To our knowledge, this is among the first evidence showing that molecular disturbances within the HP is sufficient to induce psychomotor agitation in mice. Previously, research conducted in methylazoxymethanol acetate (MAM) treated rats has implicated the HP in the control of Ventral Tegmental Area (VTA) neuronal activity and striatal dopamine release.^39^ However, the MAM model is induced during gestation and the resulting neuropathology is not restricted to the HP region, whereas our model exclusively targets the HP via stereotaxic injection of AAV in adult mice. Thus, our finding suggests a critical role of hippocampal dysfunctions might play in dysregulation of subcortical dopamine systems and the pathophysiology of schizophrenia.^40^

It is worth noting that there appear to be discrepancies in the results from AAV-shRNA knockdown and AAV-difopein expression experiments. In 14-3-3 FKO mice, the shRNA virus injection in both the HP and PFC is required to significantly curtail locomotor hyperactivity (Fig. 2). However, in wild-type mice, injection of the difopein virus into the HP alone is sufficient to induce locomotor hyperactivity (Fig. 5b). As the 14-3-3 inhibitor transgene is expressed in a variety of brain regions of the 14-3-3 FKO mice, the locomotor hyperactivity exhibited by these mice is likely caused by multiple neural circuit alterations whose relationships to each other are not yet understood. This could explain why shRNA-mediated knockdown of 14-3-3 inhibitor expression in both the HP and PFC of 14-3-3 FKO mice only partially attenuates their locomotor hyperactivity. Moreover, the requirement for knocking down difopein in both HP and PFC to improve locomotor hyperactivity suggests a potentially cooperative contribution of these two brain regions to 14-3-3 FKO mice’s hyperlocomotive behaviors. Indeed, recent research supports the idea that a direct pathway from the HP and subiculum to the PFC is critically involved in some aspects of cognitive and emotional regulation,^41,42^ and impairments in spatial working memory correlate with deficits in functional connectivity between the HP and PFC in certain mouse models of schizophrenia.^43,44^ Considering that injection of AAV-difopein in both the HP and PFC of wild-type mice did not further increase their locomotor hyperactivity (comparing to mice injected with difopein in the HP alone), a cooperative action of these two brain regions in the 14-3-3 FKO mice may involve other brain regions(s) or neural circuit(s) which are also affected by difopein expression. Nevertheless, we were somewhat surprised that injection of AAV-difopein in the PFC alone did not significantly enhance locomotor activity in wild-type mice. Recently, Kim et al.^33^ reported that a molecular disturbance in the frontal cortex is sufficient to induce locomotor hyperactivity via a cortical-to-midbrain neural circuit in mice. Based on that study, disruption of Arp2/3 activity in the frontal cortex induces progressive loss of dendritic spines, as well as promotes the formation of abnormal synaptic contacts including axo-dendritic synapses at the shaft and double synapses at dendritic spines, which may contribute to the enhancement of excitability of pyramidal cells.^33^ However, 14-3-3 proteins are involved in a variety of regulatory processes, and the precise impact of 14-3-3 inhibition on cortical neurons, synaptic properties and connectivity is yet to be defined. Thus, future studies using these different molecular manipulations (Arp2/3 vs. 14-3-3) in frontal cortex may be useful to further determine the specific pathologies in cortical neural network that can trigger abnormal locomotor behavior within a long-range circuit disturbance.

Our previous studies of the 14-3-3 FKO mice demonstrate that disruption of 14-3-3 functions gives rise to perturbed synaptic transmissions and plasticity in the forebrain. Through further electrophysiological and biochemical analyses of these mice, we identified a reduction in post-synaptic NMDA receptors.^15,30^ Thus, these observations identified a potential substrate for 14-3-3’s actions at the synapse and are consistent with other reports showing that NMDA hypofunction in the forebrain leads to schizophrenia-associated behaviors, including hyperactivity.^45,46^ Here, we determined that acute 14-3-3 inhibition in specific forebrain regions also reduces NMDA receptor levels at the PSD. Moreover, there was increased levels of NMDA receptors in the HP after knockdown of the 14-3-3 inhibitor transgene. Together, these data indicate that acute 14-3-3 manipulation directly regulates NMDA receptors at the synapse and suggests that NMDA hypofunction may be linked to the onset of schizophrenia-associated psychomotor behavior.

In the recent analyses of schizophrenia genetic data sets, two particular pathways are over-represented among the risk alleles: (1) the regulation of cytoskeleton-associated proteins and (2) the NMDA receptor complexes.^26,27,47^ Based on these bioinformatic analyses, the 14-3-3 family of proteins is likely involved in the regulation of both the actin-cytoskeleton and NMDA receptors. The regulation of actin-cytoskeleton at synapses involves the Rho GTPase/LIMK-1/cofilin signaling cascade, and 14-3-3 has been shown to bind with several key elements in this pathway.^48–51^ A more recent study further suggests that 14-3-3 facilitates the degradation of δ-catenin, which increases Rho GTPases activity, resulting in the phosphorylation and inactivation of a major actin depolymerizing factor cofilin.^52^ Consistently, a reduction in dendritic spine density has been identified in several mouse models of 14-3-3 deficiency.^15,53,54^ On the other hand, studies from our transgenic and viral-mediated 14-3-3 mouse models provided experimental evidence that 14-3-3 proteins regulate NMDA receptor levels at the synapse. 14-3-3 is known to promote surface expression of NMDA receptors in cerebellar neurons through its interaction with PKB-phosphorylated GluN2C subunits.^55^ However, it remains to be determined whether 14-3-3 proteins have similar effects on the surface expression of other NMDAR subunits, or if 14-3-3 might increase the synaptic NMDAR levels by regulating other critical steps, such as dendritic transport and synaptic localization.^56–58^ Moreover, there is evidence suggesting that actin dynamics may have a regulatory role in NMDAR placement at the synapse.^59^ It thus raises a possibility that 14-3-3 regulated actin network in synapses may also contribute to its effect on the synaptic expression of NMDA receptors. As NMDA receptor dysregulation is implicated in the pathogenesis of schizophrenia and other neuropsychiatric disorder, further dissection of the molecular pathways underlying 14-3-3-dependent modulation of NMDA receptors may provide novel insight into potential targets for therapeutic treatment of schizophrenia symptoms.

In summary, the present study provides direct evidence that 14-3-3 inhibition in specific forebrain regions leads to certain schizophrenia-associated endophenotypes. While our results collectively implicate both the PFC and HP, we determined that 14-3-3 inhibition specifically within the HP is sufficient to induce psychomotor activity. In combination with other analytical approaches, further work using the AAV-mediated gene delivery technique to target specific brain regions will determine how 14-3-3 inhibition leads to particular behavioral deficits at the molecular, synaptic and circuit levels.

## Materials and methods

### Animals

Generation of transgenic 14-3-3 FKO mice was previously described.^15,30^ Briefly, these transgenic mice express the yellow fluorescent protein (YFP) fused difopein (dimeric fourteen-three-three peptide inhibitor) using the Thy-1 promoter. Positive founder line mice were backcrossed to wild-type (WT) C57BL/6 mice for at least eight generations before being subjected to analyses. All tests were conducted during the light cycle. Animal groups were randomly assigned and surgical procedures were performed before testing. All animal procedures were carried out in accordance with the guidelines for the Care and Use of Laboratory Animals of Florida State University and approved by the Florida State University Animal Care and Use Committee.

### Viruses

All adeno-associated virus (AAV) used here were constructed and produced by the Obio Technology (shanghai) CO., LTD. The YFP-targeting shRNA sequence was selected based on a previously reported lentiviral vector, pSicoR-mCh-GFPi.^60^ The DNA fragment (ACAGCCACAACGTCTATATttcaagagaATATAGACGTTGTGGCTGT) was synthesized and cloned into an shRNA-expressing rAAV vector, pAKD-CMV-mCherry-U6-GFPi. A similar strategy was used to construct its negative control (NC) rAAV vector (pAKD-CMV-mCherry-U6-NC) that expresses a nonspecific DNA fragment (TTCTCCGAACGTGTCACGTttcaagagaACGTGACACGTTCGGAGAA). The cDNA encoding YFP-difopein was subcloned into the rAAV vector, pAOV-CaMKIIa-YFP-difopein. These viruses (AAV serotype 2/9) were then produced using the triple transfection method in HEK 293 cells and AAV titers were determining by real-time PCR.

### Stereotaxic surgery

For infections of viruses, mice were deeply anesthetized with intraperitoneal (i.p.) injection of ketamine (100 mg/kg)/xylazine (10 mg/kg). A 33-gauge needle was positioned in the frontal cortex (RC: + 2.5 mm, ML: ± 1.0 mm, DV: −1.5 to −2.0 mm brain surface, relative to bregma) and/or into the dorsal HP, CA1 (RC: −2.1 mm, ML: ± 2.0 mm, DV: −1.4 mm brain surface, relative to bregma) using a stereotaxic frame (Kopf stereotaxic instruments). Viruses (0.5 μl) were infused slowly over 3 min into the targets using a microdriver with a 10 μl Hamilton syringe.

### Behavioral tests

*Open field activity*. Mice were placed into a square open field arena (Med Associates Open Field Arena, 43.2 cm × 43.2 cm × 30.5 cm, with IR photobeam sensors) and their general activity was assessed for 30 min using Med Associates Activity Monitor software.

*Fear conditioning*. Contextual fear conditioning was performed as previously described.^30^ The contextual fear conditioning test was performed over 2 consecutive days using the Contextual NIR Video Fear Conditioning System for Mouse and Video Freeze Software (Med Associates). For training on day 1, mice were placed in the testing chamber and their freezing behavior was recorded before (3 min), during, and after (2 min) 3 footshocks (2 s, 0.50 mA, separated by a 2 min intershock interval). For testing on day 2, mice were placed back into the same chamber and their freezing behavior was recorded for 3 min.

*Sensorimotor gating*. Startle response and pre-pulse inhibition (PPI) of the acoustic startle response were assessed using Acoustic Startle Reflex package (Med Associates). The test mouse was placed in a clear Plexiglas cylinder holder within a sound attenuating cubicle, and their responses to acoustic stimuli were recorded with 62 dB background noise. Baseline startle amplitude responses were first measured during a block of ten trials consisting only of startle tone (110 dB). PPI was determined in the subsequent block which consisted of pseudo-randomized delivery of 28 trials, including four trials with startle tone only, four trials with no stimuli, four trials with pulse tone only (67, 70, 73, or 76 dB), and 12 trials with pulse paired with startle tone. PPI percentage was calculated as: [((Mean of Baseline Startle Only – Mean of Startle with Prepulse)/Mean of Baseline Startle Only) * 100%].

### Fluorescence imaging

Mice were anesthetized and transcardially perfused with 4% paraformaldehyde in 0.1 M phosphate buffer, pH 7.4 (PBS). After an overnight postfixation in the same fixative at 4 °C, the brains were then cut into 40 μm sections on a Vibratome (Leica Microsystems). Brain sections were mounted with Vectashield to retard fluorescence fading, and imaged on a fluorescence microscope using a 4× objective. To further examine the viral expression, the sagittal sections were imaged on a Zeiss laser scanning confocal microscope (Zeiss LSM 880) using a 20× objective.

### Western blot

The mouse hippocampal postsynaptic density (PSD) fractions were prepared as previously described.^15,30^ Briefly, dissected dorsal hippocampi were homogenized in TEVP buffer (10 mM Tris base, 5 mM NaF, 1 mM Na3VO4, 1 mM EDTA, 1 mM EGTA, pH 7.4) + 320 mM sucrose solution. Homogenates were centrifuged (800 × *g*) to remove nuclei and large tissue debris (brain lysate fraction). The supernatant was centrifuged at 9200 × *g* to yield the crude synaptosomal membrane fraction and the soluble fraction. The membrane fraction was subjected to hypo-osmotic shock (TEVP buffer + 35.6 mM sucrose) and centrifuged at 25,000 × *g* to yield the lysed synaptosomal membrane fraction (PSD). The PSD fractions were then sonicated and separated by SDS-PAGE and probed with specific antibodies. The relative amount of GAPDH (probed with anti-GAPDH, Life technologies, Product No. AM4300, Lot No. 00583807) was used as a loading control for quantification. Other primary antibodies used in this experiment include monoclonal antibodies against PSD95 (Abcam, Cat No. ab2723, lot No. 6G61C9), and GluN1 (NR1, Millipore, Cat. No 05–432, Lot No. 2726812), as well as polyclonal antibodies against GFP (Santa Cruz Biotechnology, Cat. No. sc-8334, Lot No. K1215), and GluN2A (NR2A, Millipore, Cat. No 07–632, Lot No. 1972322). The western blot signals were generated by incubating the membranes with fluorescently-labeled secondary antibodies (LI-COR Biosciences, Goat anti-mouse: Cat. No 926–32210, Lot No C70301–02; Goat anti-rabbit: Cat. No 926–32211, Lot No C41217–04), and acquired using the LI-COR Odyssey Infrared Fluorescent scanner. Protein densities on western blots were then analyzed and quantified with ImageJ software. Protein levels were determined by normalizing the band intensities to that of controls. All blots derive from the same experiment and were processed in parallel.

### Experimental design and statistical analysis

Viruses were stereotaxically injected into 5–6-month-old mice (male and female). For 14-3-3 FKO mice or WT mice injected with either AAV-mCherry-shRNA or AAV-mCherry-NC (negative control), open field testing was conducted 2 weeks post-injection, and the brains were collected for PSD fractionation or fluorescence imaging the following week. For WT mice injected with either AAV-CaMKIIα-YFP-difopein or AAV-CaMKIIα-YFP (negative control), open field testing was conducted 2 weeks post-injection. PPI is measured one week after open field testing, and fear conditioning was performed the following week. The brains were collected for PSD fractionation or fluorescence imaging one week after all the behavior tests is completed. Experimenter was blinded to the genotypes and treatments.

All data are presented as means ± standard error of the mean (S.E.M.) and were assessed by one-way ANOVA for comparisons using SPSS. When homogeneity of variance is violated, a Welch test is used as correction. A value of *p < 0.05 was considered to be a statistically significant difference.

## Supplementary information

Supplemental information

## Data Availability

The data that support the findings of this study are available within the paper. Additional data including confocal images, genotyping, and the behavioral programs that were generated and/or analyzed during the current study are available from the corresponding author upon reasonable request.

## Supplementary information

**Supplementary information** accompanies the paper on the *npj Schizophrenia* website (10.1038/s41537-018-0069-1).
