# Supplementary material for: Region-specific inhibition of 14-3-3 proteins induces psychomotor behaviors in mice
Source: NPJ Schizophr. 2019 Jan 14;5:1. doi: 10.1038/s41537-018-0069-1 (PMC6386769; doi:10.1038/s41537-018-0069-1)
Supplement: Supplementary file 1 — Supplemental information [file 41537_2018_69_MOESM1_ESM.pdf]

Supplementary Table 1. List of antibodies used in western blotting

	Antibody	Vendor	Catalog #	Lot #	Host	Dilution
Primary antibodies	anti-GAPDH	Life technologies	AM4300	00583807	mouse monoclonal	1:10,000
	anti-GFP	Santa Cruz Biotechnology	sc-8334	K1215	rabbit polyclonal	1:1000
	anti-NR1	Millipore	05-432	2726812	mouse monoclonal	1:1000
	anti-NR2A	Millipore	07-632	1972322	Rabbit polyclonal	1:1000
	anti-PSD95	Abcam	ab2723	6G61C9	mouse monoclonal	1:1000
Secondary antibodies	anti-Mouse	LI-COR	926-32210	C70301-02	Goat	1:10,000
	anti-Rabbit	LI-COR	926-32211	C41217-04	Goat	1:10,000

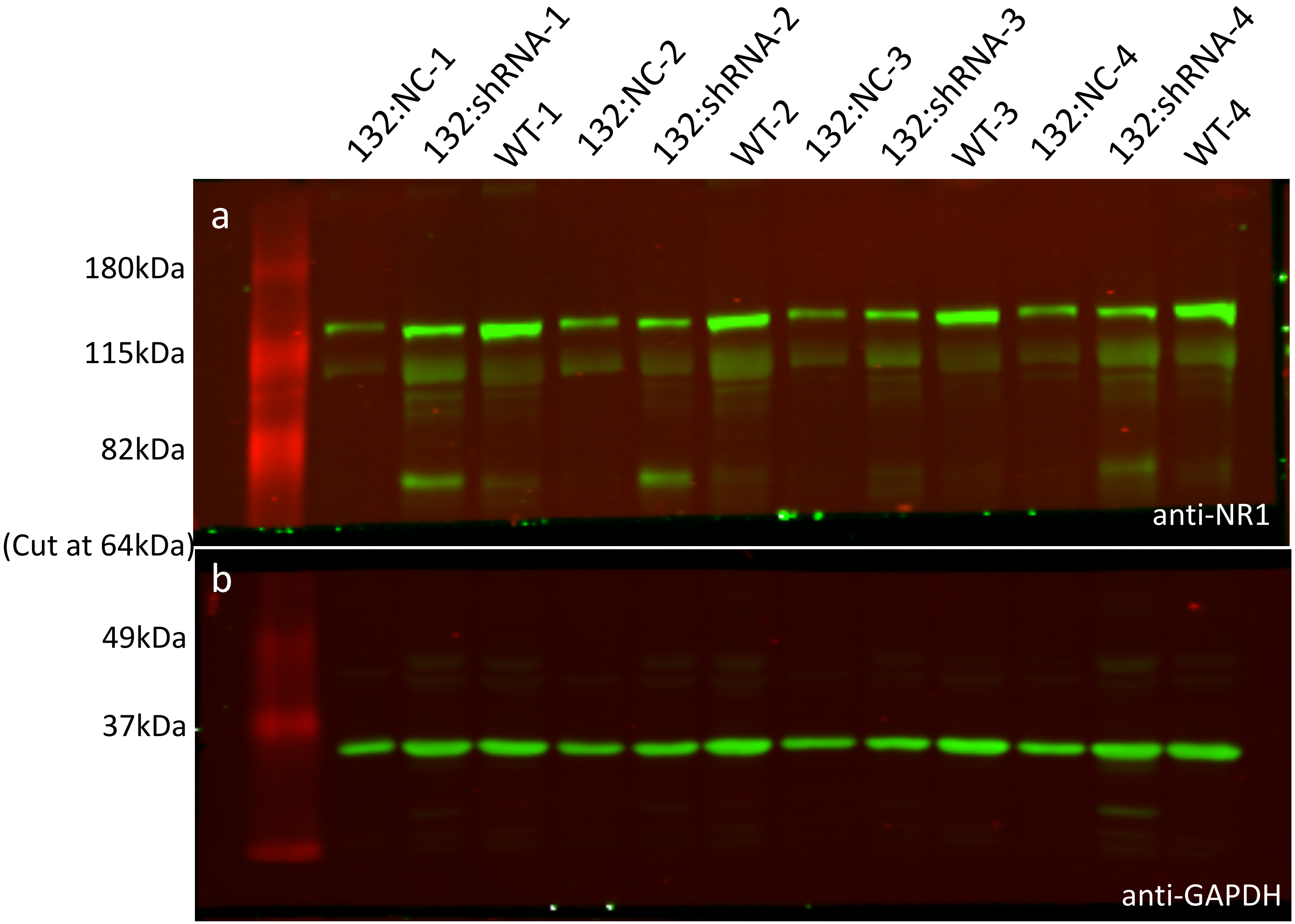

Supplementary Figure 1. Un-cropped blot #1 for Fig.3A. This membrane was cut at 64kDa after transfer. Then the top half was blotted with anti-NR1(a), bottom half was blotted with anti-GAPDH(b). (132 refer to 14-3-3 functional knock-out mice.)

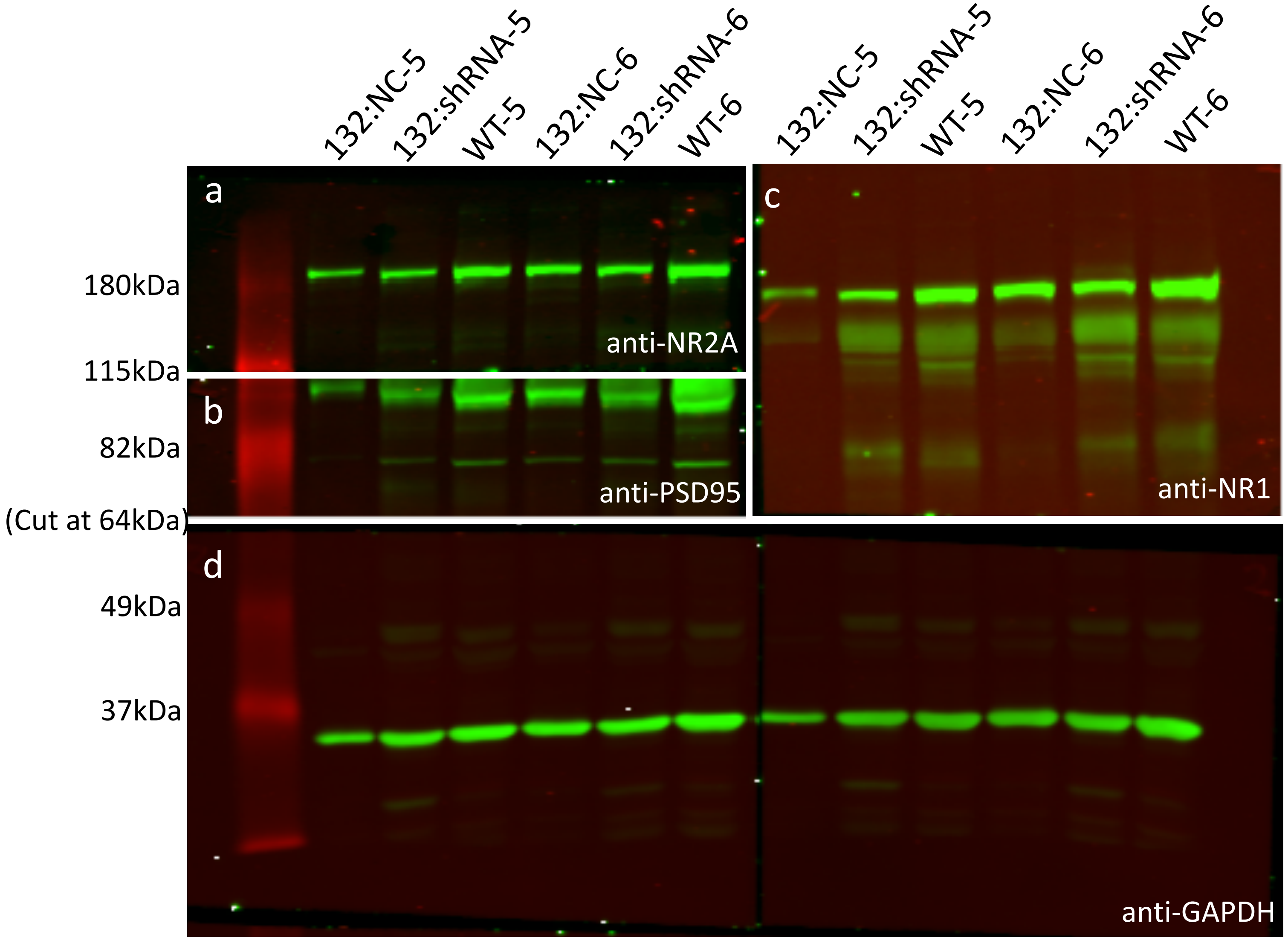

Supplementary Figure 2. Un-cropped blot #2 for Fig.3A. After transfer, this membrane was first cut in the middle, left half was then cut at 115kDa and 64kDa, right half was cut at 64kDa. Membranes were blotted with anti-NR2A(a), anti-PSD95(b), anti-NR1(c), and anti-GAPDH(d).

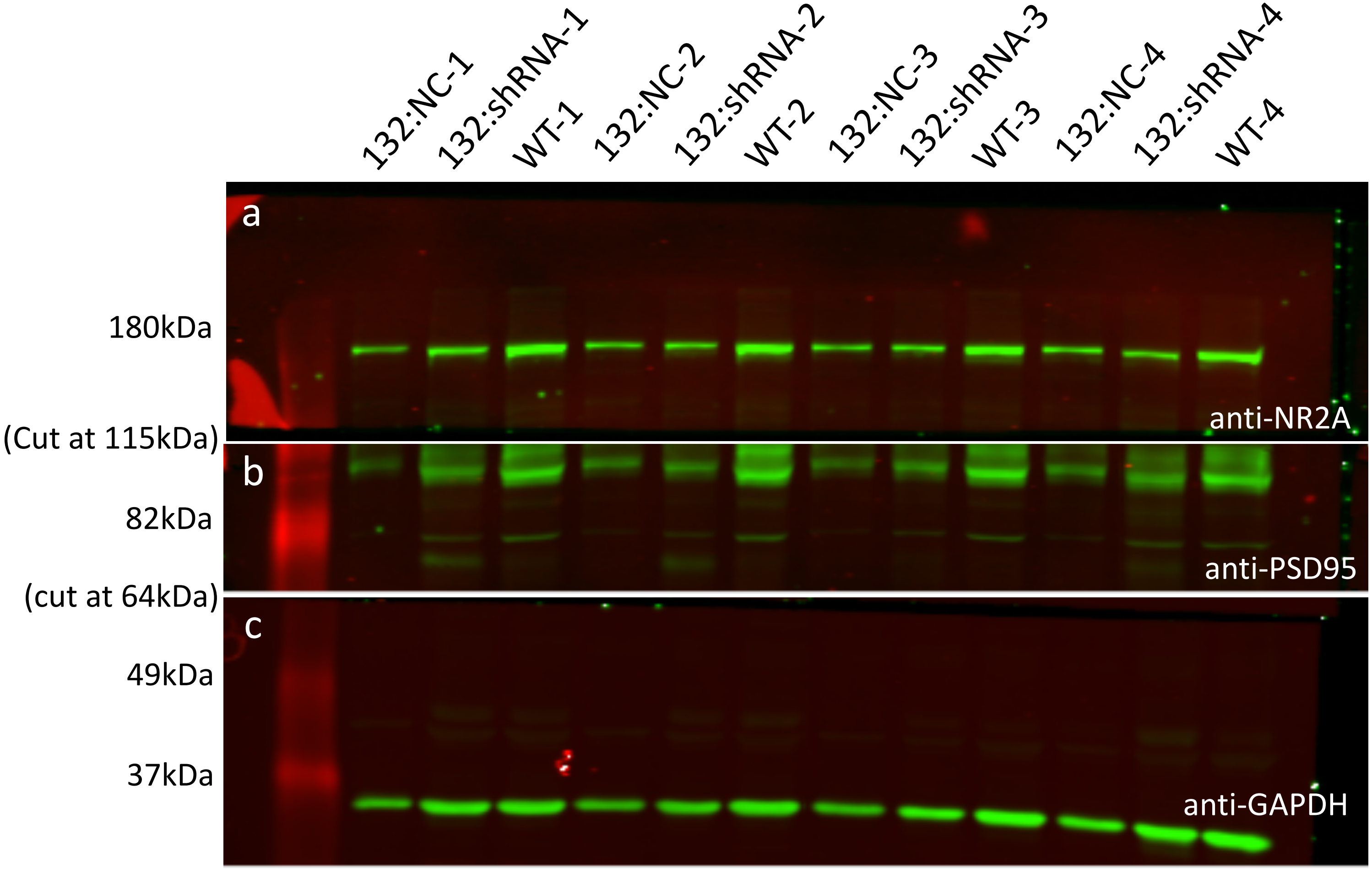

Supplementary Figure 3. Un-cropped blot #3 for Fig.3A. After transfer, this membrane was cut at 115kDa and 64kDa. Membranes were blotted with anti-NR2A(a), anti-PSD95(b), and anti-GAPDH(c).

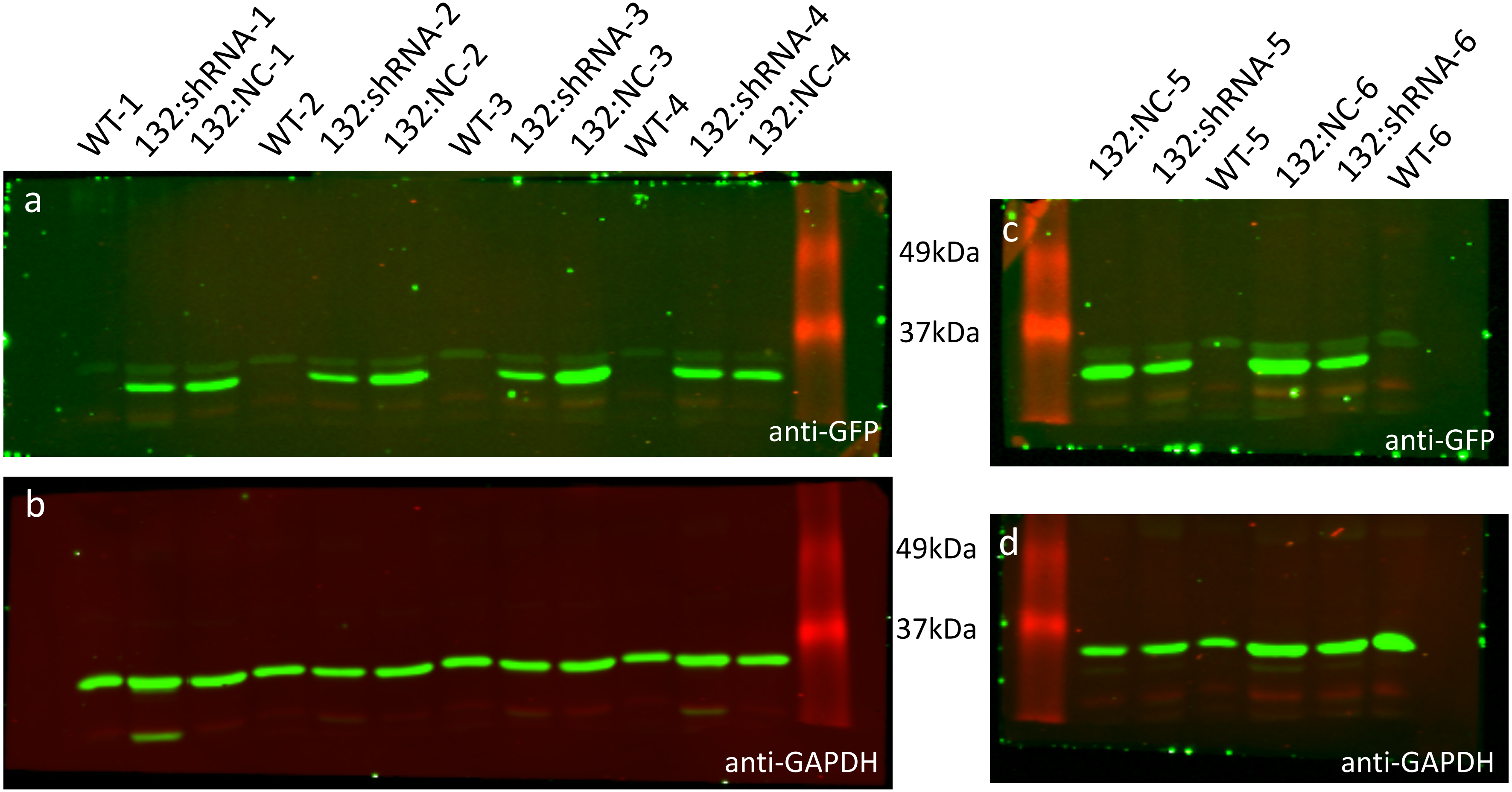

Supplementary Figure 4. Un-cropped blot #1 and #2 for Fig.3C. This membrane was cut in the at 64kDa after transfer. Membranes were first blotted with anti-GAPDH(b, d). After scanning, the membranes were stripped and re-blotted with anti-GFP(a, c).

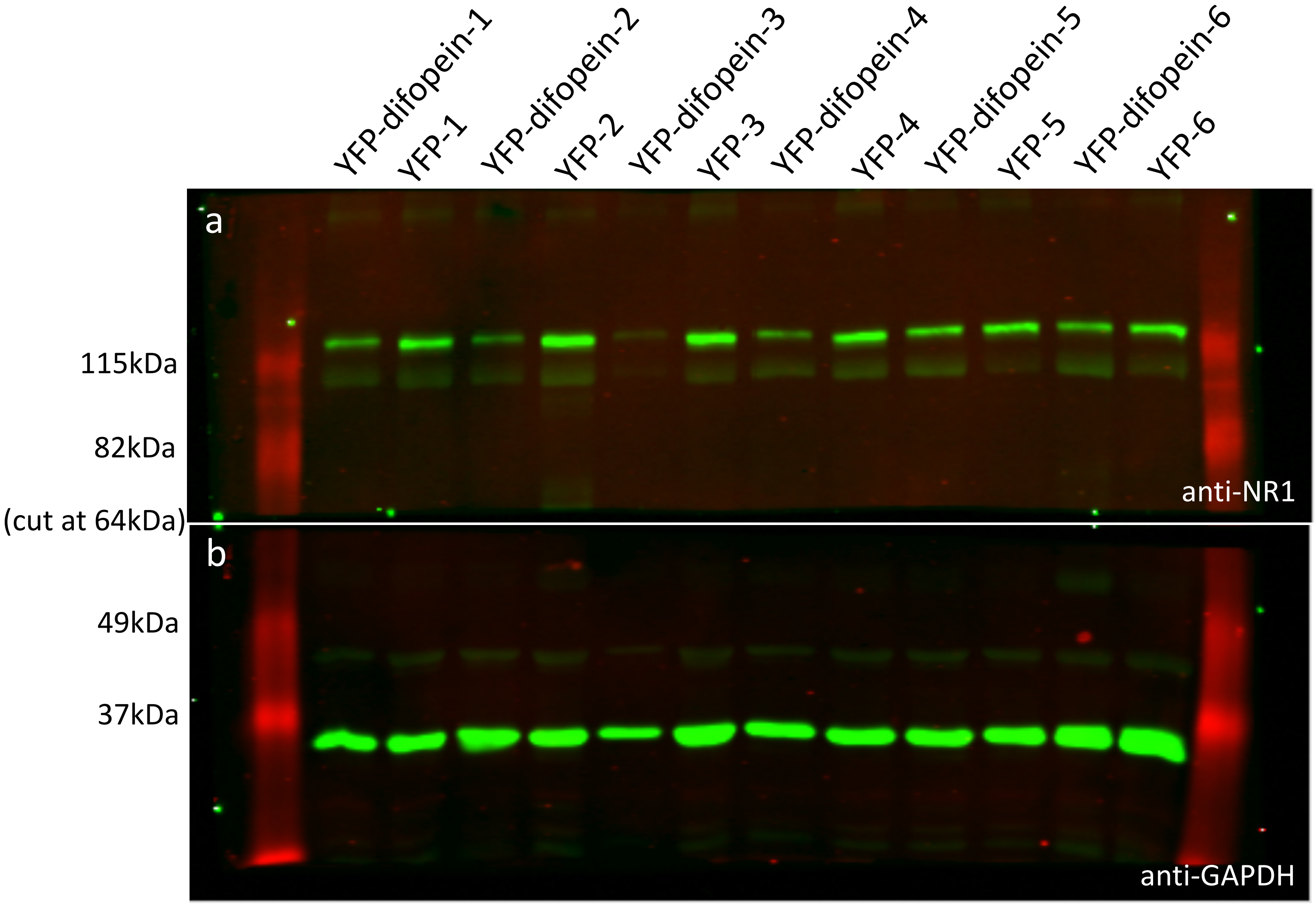

Supplementary Figure 5. Un-cropped blot #1 for Fig.6A. This membrane was cut in the at 64kDa after transfer. Membranes were blotted with anti-NR1(a) and anti-GAPDH(b).

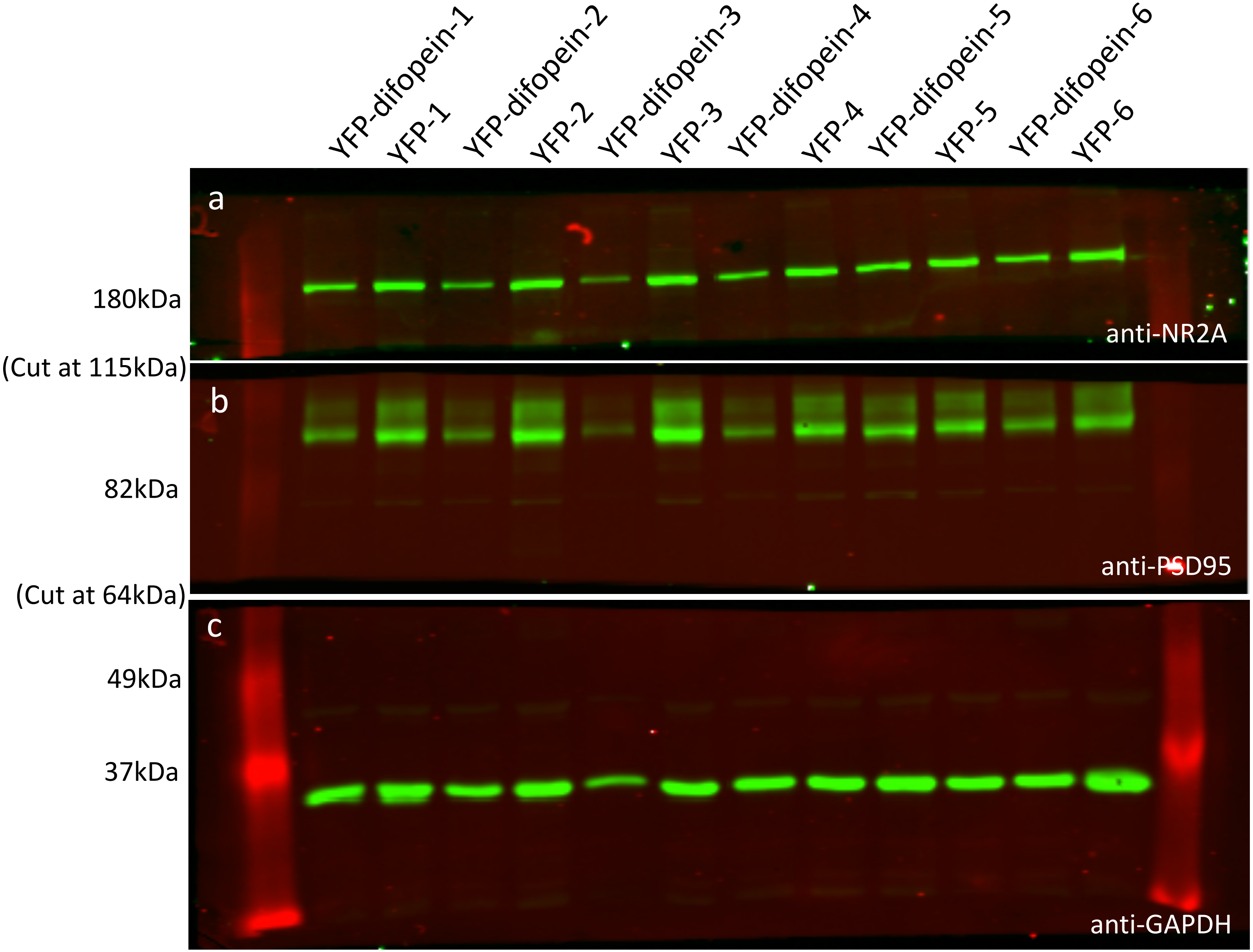

Supplementary Figure 6. Un-cropped blot #2 for Fig.6A. This membrane was cut in the at 115kDa and 64kDa after transfer. Membranes were blotted with anti-NR2A(a), anti-PSD95(b), and anti-GAPDH(c).
